# Effect of delayed entry on performance of the BACT/ALERT FAN PLUS bottles in the BACT/ALERT VIRTUO blood culture system

**DOI:** 10.1007/s10096-020-04042-z

**Published:** 2020-10-09

**Authors:** Mary Adamik, Anne Hutchins, Jasmin Mangilit, Betsy Katzin, Heather Totty, Parampal Deol

**Affiliations:** bioMérieux, Inc., Durham, NC USA

**Keywords:** Blood culture, BACT/ALERT®, VIRTUO®, Sepsis, Delayed entry, Pre-incubation

## Abstract

Delayed entry of patient blood culture samples into a microbial detection system is unavoidable at times, due to off-shift staffing or transporting samples to centralized laboratories. Pre-incubation time and temperature of blood culture bottles are the most critical factors impacting recovery and detection of microorganisms. A total of 1377 BACT/ALERT® (BTA) Fastidious Antimicrobial Neutralization (FAN® PLUS) bottles (FA PLUS, FN PLUS, and PF PLUS) were tested after delayed entry times of 24 and 36 h at 20–25 °C (room temperature, RT) prior to loading into the BACT/ALERT® VIRTUO® microbial detection system (VIRTUO). Clinically relevant organisms were inoculated into bottles with 5–84 colony forming units (CFU) per bottle, and human blood (0 to 10 mL), and then loaded into the VIRTUO. When bottles were loaded without delay, a mean time to detection (TTD) of 9.6 h was observed. For delayed bottles, the TTD reported by the VIRTUO was added to the 24-h and 36-h delay times and resulted in average time to results of 32.5 h and 42.5 h, respectively. The FAN PLUS bottles in conjunction with the VIRTUO produced acceptable results when delays up to 24 h at 20–25 °C occur in loading.

## Introduction

In 2017, the World Health Assembly (WHA) and World Health Organization (WHO) made sepsis a global health priority that causes approximately six million deaths worldwide each year [1]. The WHA resolved to improve the prevention, diagnosis, and management of sepsis. Increased mortality rates correlated with delays in diagnosis of blood stream infections (BSI) and subsequent delays in initiation of effective treatment [2]. Blood culture bottles continue to be the gold standard for isolating the infectious agent from a patient. Ideally, blood or sterile body fluids are inoculated directly into culture bottles at the patient’s bedside and then transported to a clinical microbiology laboratory without delay. However, some hospitals have microbiology labs that are closed or under-staffed during off-shift, weekends, and holidays, or, do not have the facilities or equipment to perform blood cultures in-house. Without an in-house laboratory, samples are collected and transported by courier at scheduled times to a central microbiology laboratory. During transport, delays can occur due to courier schedules and there may be temperature variations due to storage temperature or refrigeration of the samples.

Few studies have been conducted that investigate the effects of time between blood culture inoculation and loading of bottles onto a detection system on the recovery rates of microorganisms. Earlier delayed entry studies were conducted with smaller sample sizes, prior versions of the detection system BACT/ALERT® 3D (BTA 3D), and FAN blood culture bottles that contain charcoal [3–5]. The delayed entry performance of the recently launched BTA FA PLUS bottle, which contains adsorbent polymeric beads, has only been reported for a single non-fastidious organism, *Escherichia coli*, in bottles held at room temperature (RT) for 4 h, 8 h, or 12 h before loading [6]. The study described in this manuscript evaluated the performance of aerobic and anaerobic blood culture bottles seeded with a panel of microorganisms commonly found in BSIs, including fastidious bacteria, when held 24 h and 36 h at RT prior to loading into the VIRTUO. The VIRTUO is a next-generation BTA instrument that detects microbial growth and CO_2_ production by optically monitoring the reflectance of a colorimetric sensor in each bottle over time. The VIRTUO is equipped with new algorithms designed to enhance the sensitivity and specificity and deliver significantly faster TTDs for most microorganisms tested compared with the BTA 3D and BACTEC™ FX (BD Diagnostics, Franklin Lakes, NJ, USA) automated blood culture detection systems [7–9]. Two types of aerobic bottles, BTA FA PLUS and BTA PF PLUS, and anaerobic BTA FN PLUS bottles, were inoculated with a panel of clinically relevant microorganisms and loaded into the VIRTUO. Both aerobic and anaerobic bottles were tested as it is best practice to collect clinical specimens into both bottle types to increase the amount of blood drawn from a patient, which in turn leads to better recovery, as well as better detection of facultative anaerobes common in BSI [6]. This study demonstrates the performance of FAN PLUS bottles in conjunction with the VIRTUO when bottles encounter typical delay times in loading of 24 h and 36 h as reported in some clinical laboratories.

## Materials and methods

### Study design

Aerobic bottles, FA PLUS and PF PLUS (pediatric) (bioMérieux, Durham, NC), were seeded with aerobic and facultative anaerobic organisms (Table 1). Anaerobic bottles, FN PLUS (bioMérieux, Durham, NC), were seeded with anaerobic and facultative anaerobic organisms (Table 1). Testing was performed by the R&D Microbiology department at the bioMérieux, Durham, NC site.

**Table 1:** Delayed entry test panel

Bottle type	Microorganism	Blood volume (mL)				Strain(s)^d^
		0	1	4	10	
FA PLUS/PF PLUS	*Acinetobacter baumannii*^a^	x		x	x	19606, clinical isolate 102460, clinical isolate 105298
	*Candida albicans*	x		x	x	14053
	*Enterococcus faecium*	x		x	x	35667
	*Escherichia coli*	x		x	x	25922
	*Haemophilus influenzae*^c^		x	x	x	10211
	*Pseudomonas aeruginosa*^a^	x		x	x	9027, 27853
	*Neisseria meningitidis*^b,c^			x	x	13090
	*Staphylococcus aureus*	x		x	x	25923
	*Streptococcus pneumoniae*^c^			x	x	6305, 49619, clinical isolate 10076
FN PLUS	*Bacteroides fragilis*		x		x	25285
	*Clostridium perfringens*	x			x	13124
	*Enterococcus faecium*	x			x	35667
	*Escherichia coli*	x			x	25922
	*Staphylococcus aureus*	x			x	25923
	*Streptococcus pneumoniae*^c^				x	6305, 49619, clinical isolate 10076

^a^Non-fermentative organisms

^b^Temperature-sensitive organisms

^c^Organisms associated with pediatric samples [10]

^d^ATCC strain, unless otherwise noted

### Blood

Human blood from healthy willing donors was collected into blood collection tubes containing 0.35% sodium polyanetholesulfate (SPS) in 0.85% sodium chloride (Becton Dickenson, Franklin Lakes, NJ). Blood donors signed an informed consent form in accordance with company policies. The collected blood was pooled and then added aseptically to bottles on the day of collection as needed.

The FA PLUS and FN PLUS bottles were tested with blood (10 mL, recommended blood volume for adult samples), and without blood to represent sterile body fluids, which typically do not contain blood. The PF PLUS bottles were inoculated with 4 mL of blood, the recommended blood volume for the pediatric bottle. Fastidious organisms requiring blood, such as *Bacteroides fragilis* (*B. fragilis*) and *Haemophilus influenzae* (*H. influenzae*), were tested with a minimum blood volume of 1 mL, or in the case of *Neisseria meningitidis* (*N. meningitidis*), 4 mL of blood. Blood sterility was ensured by testing un-inoculated bottles containing only blood on each test date. These bottles also served as negative controls.

### Organisms

Nine microorganisms were tested in FA PLUS and PF PLUS bottles, and six microorganisms in the FN PLUS bottles (Table 1). One isolate per microorganism was tested, except for the following: *Acinetobacter baumannii* (*A. baumannii*) and *Streptococcus pneumoniae* (*S. pneumoniae*) (two additional strains), and *Pseudomonas aeruginosa* (*P. aeruginosa*) (one additional strain). Each strain was tested in triplicate per bottle type, blood volume (depending on organism growth requirement), hold time, and instrument (three VIRTUOs) using a single inoculum suspension. Repeat testing was performed for a subset of bottles seeded with *N. meningitidis* due to an instrument loading error. Organisms were selected based on clinical significance, and as representative organisms that challenge the detection technology and algorithms of the VIRTUO, such as fastidious organisms, organisms sensitive to temperature fluctuations, or low CO_2_ producers [11, 12]. Multiple strains of *A. baumannii*, *P. aeruginosa*, and *S. pneumoniae* were tested due to observed recovery failures in previous delayed entry studies [3–5]. The organisms *H. influenzae*, *N. meningitidis*, and *S. pneumoniae* were selected due to their prevalence in pediatric bacteremic patients as well as their fastidious nature and poor recovery in CSF samples when subjected to long transit times and variation in temperatures [10, 13].

Each inoculum was prepared from fresh isolates on solid media. Aerobic microorganisms were suspended in tryptic soy broth (TSB) (bioMérieux, Durham, NC) and anaerobic microorganisms were suspended in Schaedler’s broth (Accumedia, Lansing, MI) to achieve an 85–90% transmittance at 660 nm, or an equivalent McFarland value (0.30 to 0.50) on a DensiCHEK Plus (bioMérieux, Durham, NC). Each concentrated suspension was then serially diluted to a target inoculum concentration of ≤ 200 CFU/mL such that upon inoculation with 0.5 mL, each bottle would receive ≤ 100 CFU/bottle. To determine the actual number of CFUs added to the bottles, a portion of each inoculum was plated on standard media. Enumeration demonstrated the actual range was 5–84 CFU/bottle and inocula were pure. Note that anaerobic organisms were prepared, inoculated into bottles, and plated for purity and enumeration in an anaerobic chamber.

## Method

Bottles inoculated with and without blood and/or organism were pre-incubated at specified times prior to loading in the VIRTUO. Control “No Delay” bottles were loaded immediately after inoculation. Test bottles were held 24 h and 36 h at 20–25 °C (RT), as it is not recommended to incubate at 37 °C or to refrigerate bottles when there is a delay in loading, per the IFU (instructions for use) of the BTA blood culture bottles. All bottles were subcultured to solid media upon removal from the VIRTUO. Due to the autolytic nature of *S. pneumoniae*, which can lead to no growth on solid media, a latex agglutination kit (Wellcogen, Kent, UK) was also used to confirm bottles were inoculated with *S. pneumoniae*. Colony morphologies (or agglutination test results) from positive bottles were observed to ensure purity of the inoculum and/or to confirm the intended microorganism was tested. Negative bottles remained in the instrument for 5 days until declared negative, and were then subcultured (or tested by agglutination) to verify the absence of an organism. Negative bottles with growth upon subculture or with a positive agglutination test would be deemed false negatives. The TTD for all bottles was recorded by the VIRTUO software.

### Limitations

A limitation of this study is the use of seeded simulated blood cultures inoculated with a controlled amount of organism and supplemented with blood from willing healthy donors, as opposed to using clinical samples from known BSI patients.

## Results

A total of 1377 bottles were tested in this delayed entry study. When aerobic and anaerobic bottles were loaded without delay, an overall detection rate of 100% (460/460) was observed. Similarly, when bottles were held for 24 h, 100% (458/458) of bottles were detected. Less than 100% was observed with bottles held for 36 h (Table 2). A decrease in the detection rate from 100.0 to 99.6% was observed as the hold time increased from no delay and 24 to 36 h. Using a chi-square equality of proportion test, no significant difference was detected between detection rates for the no delay, 24-h, and 36-h delayed hold times (*p* value = 0.2214). When bottles were loaded without delay, a mean TTD of 13.8 h was observed. The delay times of 24 h and 36 h resulted in average TTD of 8.5 h and 6.5 h, respectively (Table 2). The observed faster TTDs for the hold times of 24 h and 36 h demonstrate microbial growth occurred at RT prior to loading. The delay time needs to be added to the TTD to determine the time to patient results as any delay in loading may lead to a delay in patient care.

**Table 2:** Overall recovery at delay times and temperatures for FA PLUS, PF PLUS, and FN PLUS

Delayed entry time (h)	Delayed entry holding temperature (°C)	Avg. TTD (h)	Hold time + Avg. TTD (h)	TTD range + hold time (h)	Detection rate % # (+)/*n*
No delay	No delay	13.8	13.8	8.5–40.5	100 (460/460)
24	20–25 °C	8.5	32.5	27.4–54.3	100 (458/458)
36	20–25 °C	6.5	42.5	37.5–65.1	99.6 (457/459)
Grand total					99.9 (1375/1377)^a^

^a^Two bottles seeded with *Acinetobacter baumannii* were negative when held at RT for 36 h. One of these bottles was a false negative

### Delayed entry performance of aerobic bottles

Aerobic FA PLUS and PF PLUS bottles were evaluated using nine aerobic and facultative anaerobic microorganisms (Table 1). An overall detection rate of 99.7% (646/648) was observed for the FA PLUS bottles tested. All FA PLUS bottles were declared positive when loaded without delay, or with a 24-h delay. Two FA PLUS bottles, seeded with *A. baumannii* and held at RT for 36 h, were declared negative at 5 days. Of the two instrument-declared negative FA PLUS bottles, one was determined to be a false negative based on subculture. This was the only false negative observed out of 1377 bottles tested. For PF PLUS, a detection rate of 100% was observed for all bottles loaded (379/379) (Table 3).

**Table 3:** Result of delayed entry on organism TTD in FA PLUS, PF PLUS, and FN PLUS bottles on VIRTUO

Organism (strain #) actual CFU/bottle	Delayed entry time (h) and holding temperature (°C)											
	No delay				24 h (20–25 °C)				36 h (20–25 °C)			
	Avg. TTD (h)	TTD range (h)	# (+)/*n*	Detectionrate (%)	Avg. TTD (h)	TTD range (h)	# (+)/*n*	Detectionrate (%)	Avg.TTD (h)	TTDrange (h)	# (+)/*n*	Detectionrate (%)
*Acinetobacter baumannii* (19606) 11 CFU, (102460) 9 CFU, (105298) 5 CFU	12.0	10.1–14.7	81/81	100	6.0	3.8–10.0	81/81	100	3.8	2.7–7.7	79/81	97.5
*Bacteroides fragilis* (25285) 62 CFU	32.7	27.5–40.5	18/18	100	24.5	20.2–30.3	17/17	100	20.2	15.4–25.8	18/18	100
*Candida albicans* (14053) 26 CFU	26.9	23.0–30.5	26/26	100	12.4	10.0–14.0	27/27	100	5.8	2.8–9.8	27/27	100
*Clostridium perfringens* (13124) 47 CFU	10.3	9.1–13.0	18/18	100	6.8	5.5–9.6	18/18	100	3.1	2.8–3.7	18/18	100
*Enterococcus faecium* (35667) 12 CFU	10.6	9.2–15.2	45/45	100	4.2	3.4–5.5	45/45	100	2.4	1.5–3.2	45/45	100
*Escherichia coli* (25922) 16 CFU	9.7	9.0–10.9	45/45	100	4.1	3.5–5.5	45/45	100	2.3	1.7–2.9	45/45	100
*Haemophilus influenzae* (10211) 26 CFU	12.5	11.7–13.3	27/27	100	10.4	9.2–12.0	27/27	100	9.3	8.5–10.0	27/27	100
*Neisseria meningitidis* (13090) 40, 50 CFU	18.8	15.7–26.2	20/20	100	18.4	16.1–22.6	18/18	100	21.3	16.6–29.1	18/18	100
*Pseudomonas aeruginosa* (*9027*) 22 CFU, (*27853*) 30 CFU	14.3	11.2–16.3	54/54	100	7.2	6.1–9.7	54/54	100	4.3	3.2–6.0	54/54	100
*Staphylococcus aureus* (25923) 25 CFU	13.2	10.0–21.2	45/45	100	9.1	5.5–16.7	45/45	100	7.0	3.8–14.0	45/45	100
*Streptococcus pneumoniae* (6305) 11 CFU,(*49619*) 84 CFU, (*10076*) 9 CFU	11.3	8.5–14.7	81/81	100	9.2	7.7–14.1	81/81	100	8.8	6.4–13.0	81/81	100

### Delayed entry performance of anaerobic bottles

Anaerobic FN PLUS bottles were tested with six microorganism species comprised of both strict and facultative anaerobes (Table 1). Seeded FN PLUS bottles demonstrated a 100% (350/350) detection rate at all delay conditions (Table 3).

### Time to detection and % detection by organism

Ten of the 11 organisms tested were detected 100% of the time regardless of the delay time: *B. fragilis*, *Candida albicans*, *Clostridium perfringens*, *Enterococcus faecium*, *E. coli*, *H. influenzae*, *N. meningitidis*, *P. aeruginosa*, *Staphylococcus aureus*, and *S. pneumoniae*. The organism *A. baumannii* was negatively impacted by a delay in loading. Overall, the performance of FAN PLUS bottles, when held at room temperature for 24 h, demonstrated 100% detection (Table 3).

## Discussion

Early detection of BSI is a critical factor in preventing septic shock mortality. A retrospective study showed that patient survival drops an average of 7.6% for every additional hour before administration of effective antimicrobial initiation within the first 6 h post-onset of septic shock hypotension [2]. Current standard of care for suspected sepsis patients includes collection of blood cultures, both aerobic and anaerobic, at the same time as administration of a broad spectrum antibiotics [14]. Early empirical therapy is critical in the treatment of patients with sepsis, but should be used for a limited time due to the potential of developing resistant bacterial strains [15, 16]. Organism identification and antimicrobial susceptibility test (AST) results are essential for antibiotic de-escalation, defined as a discontinuation or change in antimicrobial agents. De-escalation results in lower patient mortality and better antimicrobial stewardship [17, 18].

To ensure fast and accurate diagnostic results, blood culture bottles should be loaded into the detection system as soon as possible. Unfortunately, delays in loading of inoculated blood culture bottles are routinely encountered in clinical laboratories due to reduced off-shift staffing and transportation delays from satellite hospitals to a central laboratory. The majority of studies evaluating delayed entry were conducted prior to introduction of the BTA FAN PLUS bottles and the next-generation BTA detection system, the VIRTUO [4, 5]. Only one published study has evaluated the FA PLUS bottle in conjunction with the VIRTUO in a delayed entry study, but only a single organism and temperature was tested [6].

This study was designed to challenge the VIRTUO detection system using multiple aspects of the blood culture clinical workflow, such as different bottle types, blood volumes, inoculum concentrations of 5–84 CFU/bottle, and storage times defined in previous studies [19–22]. Both pediatric and adult recommended blood volumes were tested. In addition, bottles were tested without blood to represent sterile body fluids. Compared with previous delayed entry studies, the FA PLUS, PF PLUS, and FN PLUS bottles had improved detection rates and faster TTD on the VIRTUO, when loaded without delay, and held 24 h at room temperature [4, 5]. These results support an improvement in the BTA detection system consisting of the optimized FAN PLUS bottle formulations and algorithm on VIRTUO [23, 24]. When bottles were delayed 36 h, the VIRTUO demonstrated a detection rate of less than 100% for *A. baumannii*, which highlights the importance of loading bottles into the instrument as soon as possible.

Since the detection technology is based on the production of CO_2_ by the organism, low CO_2_-producing organisms were used to test the system under delayed entry conditions. Additional strains of *A. baumannii* and *P. aeruginosa* were included due to previously reported false negatives during delayed entry testing when pre-incubated at 36 °C for greater than 8 h using the predicate FAN media, containing charcoal, on the BTA 3D [3, 5]. An improvement in detection for *A. baumannii* in aerobic bottles was observed with an overall detection rate of 99.2% on the VIRTUO, with one false negative observed and one true-negative bottle, both occurring when bottles were held at RT for 36 h. The detection rate for *P. aeruginosa* was 100% for all hold times and temperatures.

In addition, three strains of *S. pneumoniae* were included as false negatives have been observed, in previous studies, when FAN charcoal bottles were pre-incubated at 36 °C for longer than 24 h [4]. All three FAN PLUS bottle types inoculated with *S. pneumoniae* demonstrated 100% detection at all hold times and temperatures when tested on the VIRTUO.

The FN PLUS bottle demonstrated a 100% (350/350) detection rate when tested at every pre-incubation condition, organism, and blood volume. The FN PLUS bottle performance reinforces the best practice of collecting patient samples into both aerobic and anaerobic blood culture bottles to have the greatest chance of detecting a wide range of organisms, in particular, facultative anaerobes such as *Staphylococcus* spp., S*treptococcus* spp., and *E. coli*, which are common in BSIs.

This paper supports the conclusions from previous studies as well as the package insert recommendations to load bottles as soon as possible, but to store at room temperature if a delay is unavoidable [4, 12, 19, 25]. Additionally, guidelines from CLSI, ASM, and Cumitech recommend delivery to the lab within 2 h of collection and, if there is a delay in loading, storage at room temperature [26–28]. When bottles are loaded immediately per the recommendations, a 100% detection rate was achieved for all FAN PLUS bottle types on the VIRTUO system.

Even though patients suspected of BSI are typically quite sick, the blood cultures collected from them are not necessarily treated with urgency [29]. Significant attention has been paid to certain aspects of blood culture such as media composition, sample volume, and detection systems methods; however, few studies have focused on the logistics of blood culture samples during transportation [30].

With the VIRTUO’s automated loading, it is easy for staff with minimal training to load bottles onto the instrument during off-shift staffing of the laboratory; bottles are placed on the conveyor and the instrument loads the bottles via a robotic arm. VIRTUO instruments could be placed at strategic locations throughout the hospital, or at point of care, where blood cultures could be loaded onto the instrument without delay. This workflow would reduce the time to patient results and subsequent identification of the microorganism, and result in a quicker time to de-escalation of empirical therapy [29]. This de-escalation in therapy not only benefits the patient with optimal care but also decreases the cost of unnecessary treatment, and reduces the risk of developing resistant organisms, which is becoming a threat worldwide [22].

Since the identity of the causative agent is not known when the patient sample is collected, it is imperative to load the bottle into the instrument as soon as possible after collection. However, when delays occur, the FAN PLUS bottles and the VIRTUO demonstrate acceptable performance when samples are held at room temperature for up to 24 h. Minimizing delays and the use of the FAN PLUS bottle paired with the VIRTUO will improve detection rates of organisms, such as *A. baumannii*, and allow faster reporting to the clinician. Additionally, FAN PLUS bottles have the capability to neutralize antimicrobials [31] allowing for the detection and recovery of microorganisms from patients already receiving antimicrobial therapy, which may account for the majority (82%) of blood draws [32]. Implementation of the BACT/ALERT detection system including FAN PLUS bottles and the VIRTUO supports the most robust and fastest time to diagnosis even when delays in loading occur, thus, improving the management of sepsis. Ultimately, the adoption of these recommended practices can improve patient care with effective, targeted therapy; decrease the overall cost of treatment; and reduce the incidence of antimicrobial resistance.
